# A multicenter, prospective study to observe the initial management of patients with differentiated thyroid cancer in China (DTCC study)

**DOI:** 10.1186/s12902-021-00871-x

**Published:** 2021-10-21

**Authors:** Jie Ming, Jing-Qiang Zhu, Hao Zhang, Hui Sun, Jun Wang, Ruo-Chuan Cheng, Lei Xie, Xing-Rui Li, Wen Tian, Tao Huang

**Affiliations:** 1 Department of Breast & Thyroid Surgery, Wuhan Union Hospital, Tongji Medical College, Huazhong University of Science and Technology, Wuhan, China; 2 Department of Thyroid Surgery, West China Hospital, Sichuan University, Chengdu, China; 3 Department of Thyroid Surgery, The First Hospital of China Medical University, Shenyang, China; 4 Department of Thyroid Surgery, China-Japan Union Hospital of Jilin University, Jilin, China; 5 Department of Head & Neck Surgery, The Tumor Hospital of Gansu Province, Lanzhou, China; 6 Department of Thyroid Surgery, First Affiliated Hospital of Kunming Medical University, Kunming, China; 7 Department of Head & Neck Surgery, Sir Run Shaw Hospital, School of Medicine, Zhejiang University, Hangzhou, China; 8 Department of Breast & Thyroid Surgery, Tongji Hospital, Tongji Medical College, Huazhong University of Science and Technology, Wuhan, China; 9 Department of General Surgery, Chinese PLA General Hospital, Beijing, China

**Keywords:** Thyroid cancer, Surgery, Lymph nodes, Guidelines, Outcomes, China

## Abstract

**Background:**

To assess the gaps between the initial management of patients with differentiated thyroid cancer (DTC) in real clinical practice and the recommendations of the 2012 Chinese DTC guidelines.

**Methods:**

This multicenter, prospective study was conducted at nine tertiary hospitals across China. Eligible patients were those having intermediate or high-risk DTC after first-time thyroidectomy. During 1 year of follow-up, comprehensive medical records were collected and summarized using descriptive statistics.

**Results:**

Of 2013 patients, 1874 (93.1%) underwent standard surgery according to the guidelines (including total lobectomy plus isthmusectomy and total/near total thyroidectomy), and 1993 (99.0%) underwent lymph node dissection; only 56 (2.8%) had postoperative complications. Overall, 982/2013 patients (48.8%) received radioactive iodine (RAI) therapy after thyroidectomy. Of all enrolled patients, 61.4% achieved the target serum thyroid-stimulating hormone level, with a median time to target of 234.0 days (95% CI: 222.0–252.0). At 1 year of follow-up, proportions of patients with excellent response, incomplete structural response, biochemical incomplete response, and indeterminate response were 34.6, 11.2, 6.6, and 47.5%, respectively; recurrence or metastasis occurred in 27 patients (1.3%). During the overall study period, 209 patients (10.4%) had at least one adverse event: 65.1% of cases were mild, 24.9% moderate, and 10.1% severe.

**Conclusions:**

This was the first large-scale prospective study of how patients with DTC in China are treated in actual practice. Initial DTC management is generally safe and adheres to the 2012 Chinese guidelines but could be improved, and the level of guideline adherence did not produce the anticipated treatment response at 1 year of follow-up.

**Supplementary Information:**

The online version contains supplementary material available at 10.1186/s12902-021-00871-x.

## Background

There are four subtypes of thyroid cancer which differ in their morphology, aggressiveness, invasiveness and gene expression profile: papillary thyroid cancer (PTC), follicular thyroid cancer (FTC), anaplastic thyroid cancer (ATC) and medullary thyroid cancer (MTC). Approximately 80–85% of all thyroid cancers are PTCs, and FTC is the second most frequent subtype accounting for approximately 10–15% of all thyroid cancers [1]. PTC and FTC are generally defined as differentiated thyroid cancers (DTC) owing to the presence of well-differentiated cells.

Globally, thyroid cancer is the ninth most common type of carcinoma [2]. In China, it is ranked eighth [3], and the annual incidence has increased markedly in both men and women since about 2000 [4, 5]. A study in Beijing reported that the thyroid cancer incidence rate increased 538.71% from 1995 to 2010, with an annual percentage change of 12.12% for both sexes [6]. A more recent global study indicated that the age-adjusted incidence of thyroid cancer in China has risen from 1.07/100,000 in 1990 to 2.17/100,000 in 2017 [7], and that over the past three decades, China has continued to record the highest thyroid cancer mortality rates worldwide [7]. A study of thyroid cancer trends in the USA from 1980 to 2009 by histotype reported that the increase in incidence over time was largely driven by PTC, with a slight increase in FTC, and relatively small changes in the incidence of other histotypes [8]. The cause of this increased thyroid cancer incidence is being investigated, though it may be owing to the more frequent use of highly sensitive thyroid diagnostic procedures and increased identification of subclinical disease [9]. Moreover, changing dietary patterns and increasing obesity have also been linked to thyroid cancer risk [10].

Within current guidelines for the diagnosis and treatment of thyroid cancer, recommendations for initial management generally comprise a combination of surgical treatment, radioactive ^131^iodine (RAI) therapy for most patients, and thyroid-stimulating hormone (TSH) suppression therapy (aimed at maintaining the TSH level within the very low or low–normal range depending on the stage of the disease) [11–14]. In 2012, China also established a set of clinical management guidelines for the clinical diagnosis and treatment of thyroid cancer [15]. These guidelines were published by Endocrine Society of Chinese Medical Association, Endocrinology Group, Surgery Branch of Chinese Medical Association, Head and Neck Oncology Committee of Chinese Association against Cancer, and Chinese Society of Nuclear Medicine. Although more recent guidelines now exist for advanced DTC [16], the 2012 guidelines remain key to implementing standardized protocols for initial disease management.

However, to date, no large-scale prospective study has observed how DTC patients are actually treated and whether thyroid surgeons treat patients according to the guideline recommendations in actual clinical practice in China. Moreover, controversies remain regarding certain aspects of diagnosis and treatment for Chinese physicians with the publication of the 2015 American Thyroid Association (ATA) guideline [14].

In 2014, we established a hospital-based thyroid cancer database and follow-up system. This system aims to collect all clinical information that may be relevant to thyroid cancer, including family history, history of external exposure, preoperative laboratory and imaging findings, intraoperative surgical details and rapid frozen sections, and postoperative therapy details, pathology reports, and laboratory and imaging findings. Using this prospectively gathered information, we sought to collect and observe the clinical data, clinical experience, follow-up and prognosis of patients with intermediate- and high-risk DTC undergoing thyroid surgery, in order to understand the status of initial DTC management in tertiary centers in China, and to determine if there is a gap between the guideline recommendations and real-world clinical practice.

## Methods

### Study Design & Patients

The institutional review board or/and ethics committee for each of the nine study sites was constituted according to the requirements of the participating location. The institutional review board or ethics committee was responsible for the initial and continuing review and approval of the clinical study in accordance with the requirements of the International Conference on Harmonization (ICH) Guideline for Good Clinical Practice (GCP) E6, and local regulatory requirements of each participating region. All patients gave informed consent in accordance with the protocol, the World Medical Association Declaration of Helsinki, the ICH GCP guideline, and applicable local regulatory requirements of each participating region. The study was registered at ClinicalTrials.gov with the identifier NCT02638077.

Enrollment began on October 31, 2014 at nine large thyroid cancer clinics and ended on July 31, 2016. The last enrolled patient was completed on August 31, 2017 after 1 year of follow-up. These nine centers represented the highest level of thyroid cancer diagnosis and treatment in China. They are in Northern (The First Hospital of China Medical University, China-Japan Union Hospital of Jilin University, Chinese PLA General Hospital), Southern (First Affiliated Hospital of Kunming Medical University), Western (West China Hospital, Tumor Hospital of Gansu Province), Eastern (Sir Run Shaw Hospital), and Central (Wuhan Union Hospital, Tongji Hospital) China. Included patients were those diagnosed with DTC (based on pre-operative examinations and post-operative pathology report) who underwent first-time thyroidectomy and were identified as intermediate-risk or high-risk for post-surgical recurrence per the recurrence risk stratification of the 2012 Chinese guideline [15]. In brief, the assessment of recurrence risk in the 2012 guideline was consistent with the three-level stratification in the 2009 ATA guidelines [17], except for high-risk group, where individuals with family history of thyroid cancer was additionally included in the Chinese guideline. In addition, since 2012 Chinese guideline was written in Chinese language, we have translated relevant information to English (See Additional file 1, Tables 1, 2 and 3). All included patients were of Chinese ethnicity. Patients with a history of thyroid surgery, who had other malignant tumors or severe organ damage (New York Heart Association classes III–IV heart failure, liver failure, respiratory failure, renal failure, etc.), a medical or psychological condition that would not permit the patient to complete the study or sign the informed consent, legal incapacity or limited legal capacity, or unwillingness to be followed up were excluded from the study.

**Table 1:** Patient characteristics and initial management

Parameter		***N*** = 2013
Age (years)	Median (range)	41.8 (13.9-88.2)
≥ 55	*n* (%)	231 (11.5)
45–55	*n* (%)	530 (26.3)
< 45	*n* (%)	1252 (62.2)
Female sex	*n* (%)	1445 (71.8)
Family history of thyroid cancer	*n* (%)	38 (1.9)
Thyroiditis	*n* (%)	20 (1.0)
Hyperthyroidism	*n* (%)	36 (1.8)
Hypothyroidism	*n* (%)	9 (0.4)
Thyroid nodules	*n* (%)	666 (33.1)
Previous thyroid hormone replacement therapy	*n* (%)	37 (1.8)
Pre-operative complications	*n* (%)	13 (0.6)
Dysphonia		5 (0.2)
Neck pain		3 (0.1)
Palpitations		3 (0.1)
Thyroid symptoms	*n* (%)	
Hoarseness		41 (2.0)
Dysphagia		13 (0.6)
Dyspnea		14 (0.7)
Cervical mass		624 (31.0)
**Preoperative evaluation**		
Ultrasound	*n* (%)	1937 (96.2)
Suspicious lymph node metastases	*n* (%)	633 (31.4)
Benign	*n* (%)	608 (30.2)
Malignant	*n* (%)	1348 (67.0)
Unknown	*n* (%)	57 (2.8)
Thyroid nodules present	*n* (%)	1937 (96.2)
Number of nodules	Median (range)	2.0 (1–9)
Nodule size, cm	Median (range)	0.97 (0.08–13.20)
FNAB	*n* (%)	738 (36.7)
Thyroid/neck lymph node abnormalities	*n* (%)	1427 (71.0)
Baseline TSH available	*n* (%)	1927 (95.7)
Baseline TSH value (mU/L)	Median (range)	2.21 (0.00–100.00)
**Surgical approach**		
Total/near-total thyroidectomy	*n* (%)	1672 (83.1)
Lobectomy + isthmusectomy	*n* (%)	202 (10.0)
Other types of thyroidectomy	*n* (%)	139 (6.9)
Lymph node dissection	*n* (%)	1993 (99.0)
Central (level VI, VII)	*n* (%)	1913 (95.0)
Lateral (level I–V)	*n* (%)	880 (43.7)
Therapeutic	*n* (%)	1279 (63.5)
Prophylactic	*n* (%)	710 (35.2)
TSH suppression	*n* (%)	1841 (91.5)
RAI after surgery	*n* (%)	982 (48.8)

*FNAB* fine needle aspiration biopsy, *TSH* thyroid-stimulating hormone, *RAI* radioactive iodine therapy

**Table 2:** Clinicopathologic characteristics

Clinicopathologic characteristics	Parameters	***N*** = 2013
Intraoperative frozen section examination	Yes	1774 (88.1)
	No	239 (11.9)
**Postoperative pathologic examination**		
Extrathyroid invasion	Yes	885 (44.0)
	No	1128 (56.0)
Multifocality	Single focus	1237 (61.5)
	Multifocality	775 (38.5)
Tumor locations	Unilateral	1368 (68.0)
	Bilateral	620 (30.8)
	Isthmus only	22 (1.1)
	Unknown	3 (0.1)
Pathologic diagnosis	Papillary	1988 (99.0)
	Follicular cancer	16 (0.8)
	Follicular and papillary	5 (0.2)
	Unknown	4 (0.2)
Diameter of largest tumor (cm)	≤ 1.0	795 (39.5)
	> 1.0–2.0	683 (33.9)
	> 2.0–4.0	323 (16.0)
	> 4.0	40 (2.0)
	Unknown	172 (8.5)
Lymph node metastasis	Yes	1657 (82.3)
	No	338 (16.8)
	Unknown	18 (0.9)
	Central (level VI, VII)	1476 (73.3)
	Lateral (level I–V)	705 (35.0)
	Pretracheal LN	586 (29.1)
	Left paratracheal LN	635 (31.5)
	Right paratracheal LN	717 (35.6)
	Prelaryngeal/delphian LN	173 (8.6)
	Upper mediastinal LN	8 (0.4)
Distant metastasis	Yes	8 (0.4)

Values are presented as *n* (%)

LN *lymph node*

**Table 3:** L-T4 dosages in patients who achieved and did not achieve target serum TSH levels

Parameter	All	Patients who achieved serum TSH target	Patients who did not achieve serum TSH target	***p*** value^**a**^
**LT-4 dosage, μg/d, Median (Range)** (*N* = 1618)	100.00 (41.20–200.00)	100.00 (48.05–194.45)	100.00 (41.20–200.00)	*0.6026*
**LT-4 dosage, μg//kg/d, Median (Range)** (*N* = 1613)	1.65 (0.51–3.70)	1.69 (0.67–3.36)	1.58 (0.51–3.70)	< 0.0001

^a^Patients who did not achieve serum TSH target vs. patients who achieved serum TSH target

*LT-4* levothyroxine, *TSH* thyroid stimulating hormone

The observation period for each patient started the day the informed consent form was signed and lasted for 1 year. During this period, as this was a non-interventional study, no treatment for patients was specified. Investigators chose the therapeutic strategy for each patient according to their hospitals’ current practice in the treatment of DTC. Long-term follow-up of prognostic data such as TSH inhibition therapy, related laboratory examinations and imaging examinations, and death due to recurrence or metastasis were obtained from medical record at the recruiting hospitals. The telephone and smartphone app were used to contact the patients to remind them to have a medical visit. At least 1 year of follow-up was completed for all enrolled patients.

### Study endpoints

The primary focus of this study was to examine whether establishing a DTC database in China could provide information on the current status of DTC treatment in China, including the level of adherence to the initial treatment of patients with DTC as recommended in the 2012 Chinese guidelines [15]. To that end, the primary study endpoints were to investigate the proportions of patients who: underwent total or near-total thyroidectomy; were treated with RAI (See Additional file 1, Table 1 for further information regarding RAI treatment criteria in 2012 guideline) after undergoing total/near-total thyroidectomy; achieved serum TSH target value; and did not achieve serum TSH target value despite treatment with TSH suppression therapy.

Secondary endpoints were the time to achieve serum TSH target value; the dosage of levothyroxine (L-T4) for patients who achieved and did not achieve serum TSH target value; Proposed TSH target values were defined according to the 2012 Chinese guideline [15] (See Additional file 1, Table 3 for English translation). the proportion of response to initial management at 1 year of follow-up (assessed per the 2015 ATA guideline [14]) in patients who underwent total or near-total thyroidectomy and RAI remnant ablation; the recurrence rate at 1 year of follow-up; and adverse events (AEs) related to L-T4 (or thyroid tablet) treatment, classified according to the Medical Dictionary for Regulatory Activities version 15.0.

### Statistical analysis

The analysis population included all patients who met the eligibility criteria and who provided informed consent. Statistical analysis was performed using case report form data obtained until all patients completed (or discontinued) the study.

Descriptive analyses were conducted according to data types. Continuous variables were presented as mean (standard deviation) if the distribution was normal, or median (range) if the distribution was skewed. The differences between groups were tested using ANOVA test or Wilcoxon rank test according to their distribution. Normality of the distribution was tested using Kolmogorov-Smirnov test. Categorical variables were summarized using frequency and percentages. Cox proportional hazards model was used to assess the association between effect factors (age, weight, L-T4) and the primary outcome. For all safety variables, the baseline value was defined as the last measurement taken prior to the initiation of treatment. Statistical significance was set at 0.05 and two sided in this study. SAS software Version 9.2 (SAS Institute, Cary, NC, USA) was used for statistical analysis.

## Results

### Database overview

**Fig. 1:**
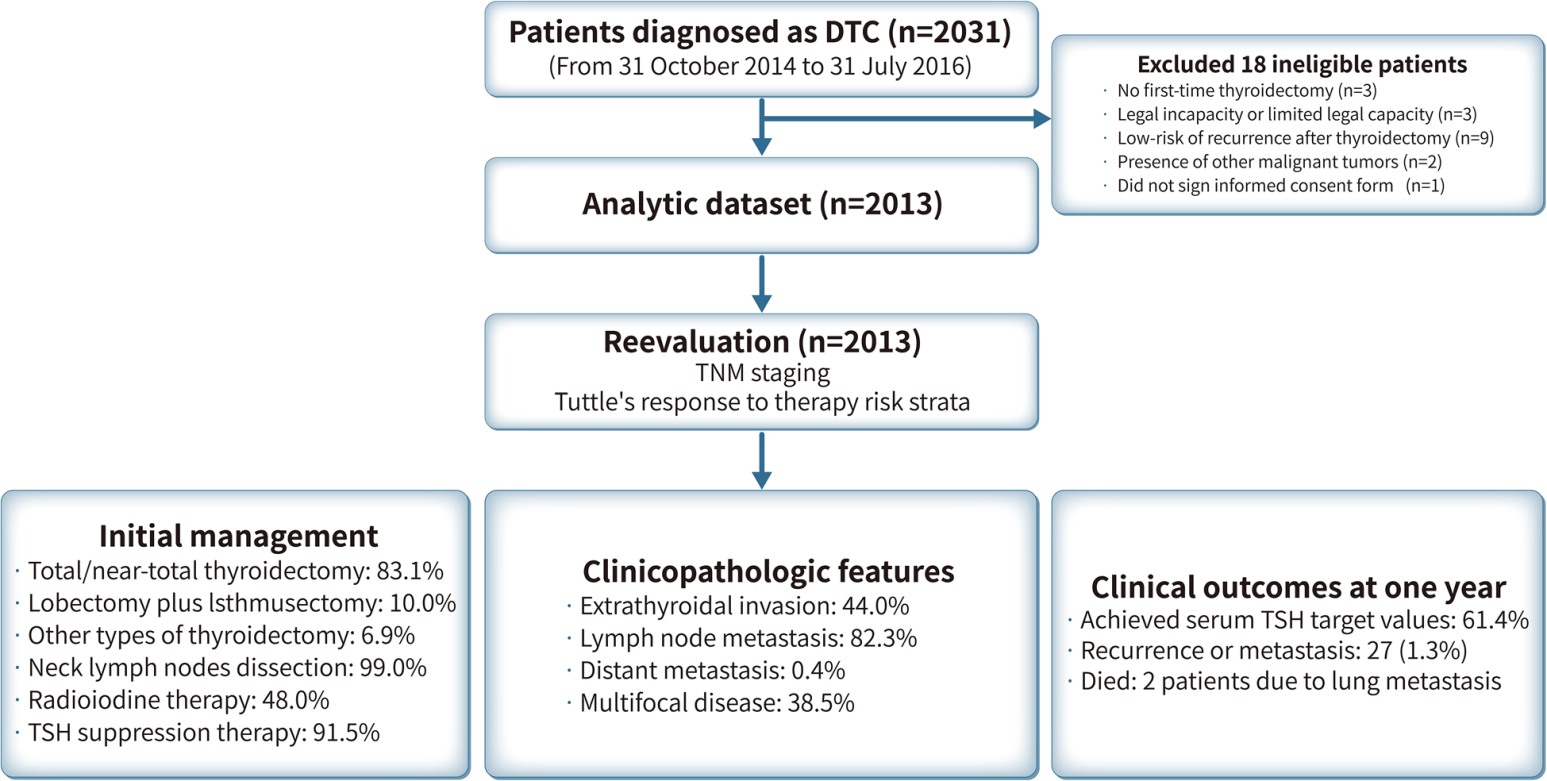
Study population. Differentiate thyroid cancer in China (DTCC study: NCT02638077)

From 31 October 2014 to 31 July 2016, 2031 patients with DTC were screened at nine clinical research centers (Fig. 1). After excluding 18 ineligible patients (no first-time thyroidectomy [*n* = 3], legal incapacity or limited legal capacity [*n* = 3], low-risk of recurrence after thyroidectomy [*n* = 9], presence of other malignant tumors [*n* = 2], and did not sign the informed consent form [*n* = 1]), 2013 patients with intermediate- to high-risk DTC were included in the analysis set. Of these, 1868 (92.7%) completed the study and 145 patients didn’t complete the study due to either poor treatment compliance (*n* = 57, 2.8%) or loss to follow-up (*n* = 88, 4.4%).

Patient characteristics are presented in Table 1. The mean age of all enrolled patients was 41.77 years (range: 13.9–88.2 years) and 1445 patients (71.8%) were females. Thirty-eight patients (1.9%) had a family history of thyroid cancer, 20 (1.0%) had thyroiditis, 36 (1.8%) had hyperthyroidism, nine (0.4%) had hypothyroidism, and 666 patients (33.1%) had thyroid nodules. A total of 37/2012 patients (1.8%) had received previous thyroid hormone replacement therapy.

The clinicopathologic characteristics of patients are shown in Table 2. The rates of extrathyroid invasion, lymph node metastasis, distant metastasis and multifocal disease were 44.0, 82.3, 0.4 and 38.5% respectively.

### Surgery

#### Thyroidectomy

The database showed that all 2013 patients underwent surgery; most patients (*n* = 1629; 80.9%) underwent bilateral total thyroidectomy or unilateral total lobectomy plus isthmusectomy (*n* = 202; 10.0%) (Table 1). Overall, 1874/2013 patients (93.1%) underwent standard surgery (including total lobectomy plus isthmusectomy and total/near total thyroidectomy) as recommended by the 2012 Chinese guidelines.

The proportion of patients with total/near-total thyroidectomy was 77.9% (619/795; 95% CI: 74.8–80.7%) for patients whose tumor size was ≤1 cm, 84.5% (849/1005; 95% CI: 82.1–86.7%) for those whose tumor size was 1–4 cm, 85.4% (35/41; 95% CI: 70.8–94.4%) for those with tumor size was > 4 cm, and 98.3% (169/172; 95% CI: 95.0–99.6%) for those whose tumor size was unknown.

#### Lymph node dissection

A total of 1993 patients (99.0%) underwent lymph node dissection during surgery; of patients with data, 1279 patients (64.3%) underwent therapeutic lymph node dissection and 710 patients (35.7%) underwent prophylactic lymph node dissection (Table 1). The extent of lymph node dissection included central neck (96.0%) and lateral neck (44.2%). For central neck lymph node dissection (*n* = 1913), the extents were pretracheal lymph node (68.8%), prelaryngeal lymph node (49.2%), left paratracheal lymph node (61.7%), right paratracheal lymph node (64.8%), and superior mediastinal lymph node (3.5%). For lateral neck lymph node dissection, the extents were compartment II, III, IV and V with proportions of 84.3, 97.8, 98.4, and 46.9%, respectively, in the left lateral neck (*n* = 503), and 88.6, 98.3, 98.9, and 49.3%, respectively, in the right lateral neck (*n* = 525).

### Post-operative therapy

#### RAI therapy after thyroidectomy

Among a total of 2013 patients undergoing thyroidectomy, 982 (48.0%) received RAI therapy. Specifically, 966 had RAI therapy with a mean administered activity of 4.08 GBq after total/near-total thyroidectomy, representing 57.8% of all patients with total/near-total thyroidectomy, and 16 patients had RAI therapy with a mean administered activity of 3.83 GBq after other types of thyroidectomy, accounting for 4.7% of all other types.

#### TSH suppression therapy

In this study, 91.5% of patients were given TSH suppression therapy, and 61.4% (1236/2013) of all evaluable patients achieved serum TSH target values. Table 3 shows dosing and TSH target value data for the patients who had available information on dosage and dosage per kilogram of body weight. The mean dosage per kilogram of body weight was significantly higher in patients who achieved the serum TSH target than in those who did not (*p* < 0.0001).

Table 4 shows proportions of patients who achieved the serum TSH target value according to different post-operative treatment scenarios, including presence or absence of RAI therapy, type of surgery, risk of recurrence, and TSH suppression therapy.

**Table 4:** Proportion of patients achieving target serum TSH levels based on post-operative treatment

Category	Target serum TSH achieved
Postoperative RAI *(N* = 982)	635 (64.7)
No postoperative RAI (*N* = 1031)	601 (58.3)
Total/near total thyroidecotomy (*N* = 1672)	1055 (63.1)
Lobectomy (*N* = 202)	110 (54.5)
High risk of recurrence (*N* = 485)	315 (64.9)
Intermediate risk of recurrence (*N* = 1528)	921 (60.3)

Values are presented as *n* (%)

*RAI* radioactive iodine, *TSH* thyroid stimulating hormone

Among the 1236 patients who achieved the serum TSH target value, the median time to achieve the serum TSH target value was 234.0 days (95% CI: 222.0–252.0). The factors influencing the achievement of the serum TSH target were analyzed by a Cox regression model (Table 5). Patients more likely to achieve the target were older (< 45 years vs. ≥ 45 years; HR 0.855; 95% CI: 0.760–0.963; *p* = 0.0100), received a higher initial dosage of L-T4 (HR 1.267 per ug/kg increase; 95% CI: 1.096–1.465; *p* = 0.0014), and weighed less (HR 0.983 per kg increase; 95% CI: 0.978–0.989; *p* < 0.0001).

**Table 5:** Effect factors related to achievement of TSH target values (Cox regression analysis)

Factors	***p***-value	HR	95% CI
Age (< 45 years vs. ≥ 45 years)	0.0100	0.855	0.760–0.963
Weight	< 0.0001	0.983	0.978–0.989
First dosage of L-T4 (μg/kg/d)	0.0014	1.267	1.096–1.465

HR > 1 indicates the factor is favorable for achieving serum TSH target values

*CI* confidence interval, *HR* hazard ratio, *TSH* thyroid-stimulating hormone

### Patient follow-up

#### Post-operative evaluation

The postoperative TNM staging was performed for all patients in the study according to both the seventh and eighth editions of the American Joint Committee on Cancer (AJCC) staging manual (15, 17). As shown in Table 6, 1313/2013 patients (65.2%) were stage I, 16/2013 (0.8%) were stage II, 336/2013 (16.7%) were stage III, and 342/2013 (17.0%) were stage IV according to the seventh edition of AJCC. If the staging was categorized according to the eighth edition of AJCC, 1845/2022 patients (91.7%) categorized as stage I, 135/2011 (6.7%) as stage II, 31/2011 (1.5%) as stage III, and none as stage IV.

**Table 6:** Post-operative evaluation

Parameter	***N*** = 2013
TNM staging (AJCC seventh edition), n (%)	2013
I	1313 (65.2)
II	16 (0.8)
III	336 (16.7)
IV A	342 (17.0)
IV B	1 (0.0)
IV C	5 (0.2)
TNM staging (AJCC eight edition), n (%)	2011
I	1845 (91.7)
II	135 (6.7)
III	31 (1.5)
Missing	2 (0.1)
Recurrence risk stratification, n (%)	2013
Intermediate	1528 (75.9)
High	485 (24.1)
Side-effect risk stratification for TSH suppression therapy, n (%)	1984
Low	1109 (55.9)
Intermediate	640 (32.3)
High	235 (11.8)
Missing	29 (1.5)

Values are presented as *n* (%)

*TNM* tumor/node/metastasis, *AJCC* American Joint Committee on Cancer

Of the 2013 evaluable patients, 1528 (75.9%) were at an intermediate risk and 485 patients (24.1%) were at a high risk of recurrence. Of these, 1984 patients had data available for side-effect risk stratification for TSH suppression therapy; the majority of patients were at a low risk (1109/1984, 55.9%).

#### Long-term outcomes

Outcomes after 1 year of follow-up are shown in Table 7. Notably, 61.4% of patients achieved serum TSH target values. Moreover, recurrence or metastasis occurred in 27 patients (1.3%) and two patients died due to lung metastasis. Table 7 also shows response to initial therapy at 1 year of follow-up for patients with total thyroidectomy and RAI ablation. Of the 966 patients with sufficient follow-up data, 31.5% were classified as having excellent response to treatment.

**Table 7:** Clinical outcomes and response to initial therapy at one year of follow-up

Clinical outcomes at one year of follow up		***N*** = 2013
Achieved serum TSH target values		1236 (61.4)
	Thyroidectomy with RAI (*n* = 982)	635 (64.7)
	Thyroidectomy without RAI (*n* = 1031)	601 (58.3)
	Total/near-total thyroidectomy (*n* = 1672)	1055 (63.1)
	Lobectomy (*n* = 202)	110 (54.5)
	Intermediate risk (*n* = 1528)	921 (60.3)
	High risk (*n* = 485)	315 (64.9)
Recurrence rate at 1-year follow-up	Total (*n* = 2013)	27 (1.3)
TSH achieved target values	Yes (*n* = 1236)	16 (1.3)
	No (*n* = 693)	11 (1.6)
	Unknown (*n* = 84)	0
Recurrence risk stratification	Intermediate risk (*n* = 1528)	19 (1.2)
	High risk (*n* = 485)	11 (1.6)
**Response to initial therapy for patients with total thyroidectomy and RAI ablation at one year of follow-up**		***N*** **= 966**
	Unknown	147 (15.2)
	Data available	819 (84.8)
	Excellent	258 (31.5)
	Biochemical incomplete	56 (6.8)
	Structural incomplete	88 (10.7)
	Indeterminate	417 (50.9)

Values are presented as *n* (%)

*HR* hazard ratio, *RAI* radioactive iodine therapy, *TSH* thyroid-stimulating hormone

In our study, only 56 patients (2.8%) had any case of postoperative complications, and most of these were temporary. For instance, 26 patients had recurrent laryngeal nerve (RLN) paralysis, among them, 23 (88.5%) were transient while three (11.5%) were permanent. Hypoparathyroidism occurred in 33 patients (1.6%); among these, 31 cases (93.9%) were transient while only two (6.1%) were permanent.

#### Safety

During the overall study period, a total of 209 patients (10.4%) had at least one AE. The severity of these was categorized as mild for 136 patients (65.1%), moderate for 52 patients (24.9%), and severe for 21 patients (10.1%), indicating that initial management was safe for most patients in this study.

There were 57 patients (2.8%) with AEs related to L-T4, such as palpitations (0.7%), hypothyroidism (0.8%), hypocalcemia (0.6%) and osteoporosis (0.6%). Eleven patients (0.5%) had AEs related to thyroid tablets, including 10 with hypothyroidism (0.5%) and one patient (< 0.1%) with hypocalcemia.

## Discussion

This analysis of data from a multicenter, prospective study in geographically spread out nine tertiary centers in China provides an insight into the real-world status of initial management of thyroid cancer following the 2012 publication of the Chinese thyroid cancer guidelines. Our results indicate that 93.1% of patients underwent standard surgery according to the guidelines and almost all (99.0%) received lymph node dissection, while only 2.8% had postoperative complications. At 1 year of follow-up, however, only slightly more than one third of patients had excellent response.

The current Chinese medical system does not assign a primary physician to patients, instead allowing patients to choose their preferred hospital. This results in high mobility of patients between hospitals, making it very difficult to follow up DTC patients in a real-world practice. This database provides important advantages in an area where there is a paucity of reliable clinical information. Patients’ baseline characteristics, intraoperative findings, and follow-up data are well documented and provide sufficient resources for analysis. In typical clinical practice in China, the importance of intraoperative findings is emphasized, as the surgeon relies on them to determine appropriate surgical methods, including the scope of thyroidectomy, whether lymph node dissection is therapeutic or preventive, and the scope of lymph node dissection. Compared with national databases initiated in other countries, such as the USA [18], Italy [19], and Denmark [20], this Chinese database is able to provide a high degree of detail in terms of key clinical information such as lymph node involvement. In contrast, lymph node data in current US databases are commonly simplified to N0/N1a/N1b, without the corresponding details of number, size, or presence of extranodal extension [18], and around one third of Danish database records are missing clinical N-stage status entirely [20].

The database has yielded useful information to date in terms of initial and post-operative management of DTC, and longer-term outcomes. While the initial management of DTC was conducted generally in accordance with the 2012 Chinese guideline recommendations, there are still areas that need to be improved, even in these high-volume tertiary centers. Among these is the continued practice of non-standard surgical types. Prior to the publication of the 2012 guideline, more than 10 types of thyroidectomy were practiced in clinics in China, including radical thyroid operation, thyroid mass resection, unilateral thyroidectomy, partial lobectomy, subtotal lobectomy, near-total lobectomy, and total lobectomy [21–29]. The 2012 guideline recommended that total/near-total thyroidectomy or thyroid lobectomy plus isthmusectomy should be considered for DTC patients [15]. However, due to controversies around the extent of surgery in China and a lack of Chinese data, the rating for this recommendation was only C. Our results demonstrated that 6.9% of patients underwent non-standard surgery, including total lobectomy plus isthmusectomy plus contralateral partial lobectomy, unilateral total lobectomy, isthmusectomy, and unilateral partial lobectomy. Despite the overall good compliance with the guidelines in our tertiary hospitals, non-standard surgeries may persist in greater proportions in lower-volume sites and smaller hospitals across China. We can speculate that, in clinical practice in China, doctors require time to develop their understanding of DTC, with preference for treatments that minimize local recurrence and metastasis. Conversely, our study reports a rate of lymph node dissection as high as 99.0%, with a low complication rate and, importantly, shows that neck dissection added to thyroidectomy was safe when compared to the data from other countries cited in the 2012 Chinese guideline (rates of RLN injury and permanent hypoparathyroidism were 4.3 and 2.2%, respectively) [15]. The reason may be that all of our thyroid surgeries were conducted by high-volume surgeons in tertiary hospitals.

Post-operative management of DTC involves administration of RAI [30], which has been shown to reduce both recurrence and death rates in DTC patients [31], and TSH suppression therapy, although data correlating TSH suppression with survival outcomes are equivocal [32]. Furthermore, in recent years, the new staging systems and prognostic tools suggested more conservative management of low- and intermediate-risk patients, indicating less extensive surgery and more use of RAI therapy [33]. In this study, RAI therapy was used in 966/1672 (57.8%) patients with total/near total thyroidectomy, and 16 patients with other types of thyroidectomy, at mean administered activities of 4.08 and 3.83 GBq, respectively. This is broadly consistent with the 2012 Chinese guideline, which recommends RAI administered activities of 3.7–7.4 GBq for intermediate/high-risk patients [15]. Most contemporary clinical trials have employed RAI doses between 30 mCi (1.1 GBq) and 100 mCi (3.7 GBq) [34]. However, controversy remains regarding exact dosing, with higher RAI doses associated with immediate, transient side effects as well as long-term consequences [35], and also around the indications for RAI [31, 36]. The preferred drug for TSH suppressive therapy is L-T4 [37]; indeed, L-T4 is specified in the guidelines as the first choice for TSH suppression therapy [15], and all 1841 patients receiving post-operative TSH suppression therapy in our analysis were treated with L-T4. Before the 2012 Chinese guideline was published, there was no consensus on the treatment goals of TSH suppression therapy and the TSH target value was unclear. Management of TSH suppression therapy appears to have subsequently improved, with “double risk-adapted stratification” recommended to establish the TSH target value for patients undergoing TSH suppression therapy [15]. However, only 61.4% of enrolled patients achieved the serum TSH target in this study. Dosage of L-T4 per kilogram of body weight was a favorable factor associated with reaching the serum TSH target.

Both initial management and L-T4 treatment were generally safe among intermediate- and high-risk DTC patients at our study centers, with few AEs. However, despite overall good adherence to the guidelines, response to initial management at 1 year of follow-up was less positive than expected. Although previous long-term follow up studies have shown that DTC patients have excellent prognosis after initial management [38–41], the rate of excellent response achieved by patients in this study was only 31.5% among the 819 patients with total/near-total thyroidectomy, postoperative RAI therapy, and evaluable response to therapy. Moreover, more than half of the patients were categorized as indeterminate response, indicating that a lot of patients should have had closer attention during follow-up. Further subgroup analysis in future studies is warranted to identify the determinants of these outcomes.

It is also worth noting that only 37% of DTC patients in this study had pre-surgical FNA use, which is higher than the estimate of 32% observed in the US [42], but lower than the estimate of 41-73% across different regions of Belgium [43]. Although ATA and other Associations recommend FNA as an important preoperative diagnostic tool for thyroid nodules, it is not widely accepted in China, and some hospitals use intraoperative frozen section for diagnosis. The relatively low rate might be explained by the fact that ultrasound guided FNA started late in China [44] and many patients choose to undergo further diagnostic procedures only when adverse ultrasound findings are present.

However, we also recognize that there are some disadvantages to the system. For example, the 2012 Chinese guideline recommends that postoperative TNM staging should be performed for all patients with DTC according to the seventh edition of the AJCC staging manual [15]. However, in 2017, the eighth edition of AJCC cancer staging was published [45]; thus, all enrolled patients in our study were evaluated using both editions. This resulted in more patients classified as stage I and fewer as stage IV when evaluated per the eighth edition, ultimately resulting in more patients being recognized as having an excellent prognosis according to the updated classification. Another complication is that in China, the laboratories and imaging examinations for preoperative evaluation of the thyroid gland are different in each clinic; this lack of standardization and the fact that there is no mandatory requirement for treatment and follow-up, mean that data loss is inevitable, potentially confounding the data. Furthermore, at present, the patient information entered in this database corresponds only with the DTC patients selected by the nine medical centers involved, which implies selection bias, and reduces the generalizability of our results. Additionally, as patients of this study dated back to 2014 to mid-2017, when the most updated WHO classification (2017) was just released, information on specific pathological diagnosis is limited. Therefore, detailed categorization as Hurthle cell carcinoma cannot be obtained. A further limitation relates to the still short follow-up of this study, which did not enable complicated analyses, especially comparison between the two groups using survival analysis. However, as the follow-up of this study is continuing, more results can be expected. Nonetheless, despite these issues, we consider that the details provided by our database provide much-needed insight into how thyroid cancer is treated in China today.

In conclusion, this was the first large-scale prospective study conducted to observe how thyroid surgeons in China treat DTC patients in real-world practice. The results show that in the years immediately following the adoption of guidelines for treatment of DTC, the initial management of Chinese DTC patients generally adhered to the guidelines, but that several areas remain to be improved, even in high-volume tertiary centers. The observed level of guideline adherence in our study did not produce the anticipated level of treatment response at 1 year of follow-up. Continued follow-up and analysis of the patient cohort in this study is currently ongoing to assess the efficacy of the different treatment strategies and the practicality of the Chinese guidelines more accurately on DTC. Importantly, the database provides guidance for disease management, supports decision making in clinical practice and provides evidence for strategies for managing Chinese patients with DTC.

## Supplementary Information

**Additional file 1.**

## Data Availability

As this is a retrospective study using existing Electronic Medical Records data, original individual patient data are not accessible in accordance with Chinese data privacy regulations. Aggregated data are accessible upon request, with the permission of all authors.

## Abbreviations

AE: Adverse event
AJCC: American Joint Committee on Cancer
ATA: American Thyroid Association
ATC: Anaplastic thyroid cancer
CI: Confidence interval
DTC: Differentiated thyroid cancer
EC: Ethics committee
FTC: Follicular thyroid cancer
IRB: Institutional review board
MTC: Medullary thyroid cancer
PTC: Papillary thyroid cancer
RAI: Radioactive ^131^iodine
TNM: Tumor/node/metastasis
TSH: Thyroid-stimulating hormone
