# Supplementary material for: A multicenter, prospective study to observe the initial management of patients with differentiated thyroid cancer in China (DTCC study)
Source: BMC Endocr Disord. 2021 Oct 21;21:208. doi: 10.1186/s12902-021-00871-x (PMC8529744; doi:10.1186/s12902-021-00871-x)
Supplement: Supplementary file 1 — Additional file 1. [file 12902_2021_871_MOESM1_ESM.docx]

**Additional file 1.** Translation of key information of 2012 Chinese DTC guideline – “the Diagnosis and Treatment of Thyroid Nodules and Differentiated Thyroid Cancer”

**Table 1.** ^131^I treatment recommendation for DTCC patients according to TNM staging ^a^

| **TNM Stages** | | **Recommended ^131^I Strength** | **RAI ablation**  **recommended** |
| --- | --- | --- | --- |
| T1 | 1 cm or less, intrathyroidal or microscopic multifocal | E | No |
|  | 1–2 cm, intrathyroidal | I | Selective use^b^ |
| T2 | >2–4 cm, intrathyroidal | C | Considering use^b^ |
| T3 | >4 cm |  |  |
|  | <45 years old | B | Yes |
|  | ≥45 years old | B | Yes |
|  | Any size, any age, minimal extrathyroidal extension | I | Selective use |
| T4 | Any size with gross extrathyroidal extension | B | Yes |
| Nx, N0 | No metastatic nodes documented | I | Selective use |
| N1 | Metastatic nodes documented |  |  |
|  | <45 years old | C | Considering use |
|  | ≥45 years old | C | Considering use |
| M1 | Distant metastasis present | A | Yes |

^a^ This is the English translation of the corresponding section of the 2012 Chinese guideline (Reference 15, Page 11, Table 5). Translation was performed by the authors.

^b^ “Selective use” means RAI ablation is neither recommended nor against to use for this entire subgroup because of either conflicting or inadequate data. “Considering use” means RAI ablation is recommended to use for this entire subgroup but not required.

**Table 2.** Recommendations for clinical practices according to 2012 Chinese DTC guideline

| **Clinical practices** | **Recommendations from 2012 Chinese DTC guideline** |
| --- | --- |
| Fine-needle aspiration cytology | 1. FNA is recommended for all thyroid nodules > 1 cm except the following cases: 1) "hot nodules" with spontaneous uptake function confirmed by thyroid radionuclide imaging; 2) nodules suggestive of pure cystic on ultrasound; 3) nodules that have been highly suspected to be malignant based on ultrasound images. 2. If thyroid nodules is < 1 cm, routine FNA is not recommended. However, ultrasound-guided FNA can be considered if the following conditions exist: 1) ultrasound suggests malignant signs of nodules; 2) with abnormal ultrasound images of cervical lymph nodes; 3) history of cervical radiation exposure or exposure to radiation contamination in childhood; 4) history or family history of thyroid cancer or thyroid cancer syndrome: 5) positive ^18^F-FDG PET imaging; and 6) with abnormally elevated serum Ct (calcitonin) levels. |
| Lymph node dissection | 1. It is recommended to perform ipsilateral central lymph node dissection under the condition of effective preservation of the parathyroid gland and recurrent laryngeal nerve. (This recommendation is based on the fact that 28%-33% of cervical lymph node metastases cannot be detected on preoperative imaging and intraoperative examination but can be diagnosed after prophylactic central lymph node dissection, which will change the stage and postoperative management of DTC.) 2. Lateral neck Lymph node dissection is recommended for DTC patients with clinical cervical non-central lymph node metastasis (cN1b). |
| Risk-stratification for side effects of TSH-suppressive therapy | 1. low risk is considered with all the following conditions: (1) young patients; (2) asymptomatic; (3) without cardiovascular disease; (4) without arrhythmia; (5) without symptoms or signs of adrenergic receptor activation; (6) without risk of cardiovascular disease; (7) without concomitant disease; (8) premenopausal women; (9) normal bone mineral density; (10) without risk factors for osteoporosis. 2. Intermediate risk is considered with any of the following conditions: (1) middle-age patients; (2) with hypertension; (3) symptoms or signs of adrenergic receptor activation; (4) smoking; (5) presence of cardiovascular disease risk factors or diabetes; (6) perimenopausal women; (7) osteopenia; (8) presence of osteoporosis risk factors. 3. High risk is considered with any of the following conditions: (1) with clinical heart disease; (2) elderly patients; (3) postmenopausal women; (4) Simultaneous present with other serious diseases. |

**Table 3.** Postoperative TSH suppression targets for DTC patients based on two-risk assessment (TSH unit: mU/L) according to 2012 Chinese DTC guideline

|  | | **Recurrence risk** | | | |
| --- | --- | --- | --- | --- | --- |
|  |  | **Initial management**  **(Within 1 year after surgery)** | | **Long-term management** | |
|  |  | Intermediate- and high-risk | Low risk | Intermediate- and high-risk | Low risk |
| **Risk for side effects of TSH-suppressive therapy** | Intermediate- and high-risk | <0.1 | 0.5~1.0 | 0.1~0.5 | 1.0～2.0  (5~10 years) ^a^ |
|  | Low risk | <0.1 | 0.1~0.5 | <0.1 | 0.5～2.0  (5~10 years) ^a^ |

^a^ Only thyroid hormone supplementary therapy is sufficient if disease-free after 5-10 years.
